# Cutaneous Changes During Pregnancy: A Comprehensive Review

**DOI:** 10.7759/cureus.69986

**Published:** 2024-09-23

**Authors:** Shreya N Gupta, Bhushan Madke, Samyak Ganjre, Sugat Jawade, Ambika Kondalkar

**Affiliations:** 1 Dermatology, Venereology and Leprosy, Jawaharlal Nehru Medical College, Datta Meghe Institute of Higher Education & Research, Wardha, IND; 2 Dermatology, Venereology and Leprosy, Shri Shankaracharya Institute of Medical Sciences, Durg, IND; 3 Dermatology, Venereology and Leprosy, Datta Meghe Medical College, Nagpur, IND

**Keywords:** cutaneous changes, face and trunk, hyperpigmentation, pregnancy, striae gravidarum

## Abstract

Pregnancy induces various physiological changes in a woman's body that significantly impact the skin involving the face and trunk. This comprehensive review explores the cutaneous changes in these regions, driven by hormonal, mechanical, and immunological factors. Physiological changes such as hyperpigmentation, striae gravidarum, and vascular alterations are discussed in addition to pathological conditions like acrochordon (skin tags), pemphigoid gestationis, impetigo herpetiformis, and intrahepatic cholestasis of pregnancy. Understanding these changes is essential for healthcare providers to offer appropriate reassurance and management to expectant mothers. This review provides insights into dermatological changes on the face and trunk during pregnancy to contribute to better clinical care and support future research.

## Introduction and background

Pregnancy is a complex physiological state characterised by significant hormonal, metabolic, and immunological changes which can profoundly affect a woman's body. These changes are essential for supporting the growing fetus, preparing the body for childbirth, and reflecting the health of both mother and baby. Among the various physiological alterations that occur, cutaneous changes and often the associated symptoms are a cause of concern for an expecting mother as they are outrightly noticeable [1]. The skin being the body's largest organ, is highly susceptible to hormonal fluctuations and mechanical stretching accompanying pregnancy. These changes can manifest in various ways which consist of common conditions to more complex dermatological issues that may require medical attention. Understanding these changes is crucial for comforting and reassuring pregnant women and guiding healthcare providers in managing and mitigating potential complications [2].

There are several important reasons for studying cutaneous changes during pregnancy. Firstly, many of these changes are distressing for women, affecting their self-esteem and overall well-being. Secondly, some cutaneous manifestations are harmless, but others can indicate underlying systemic conditions that might pose risks to the mother and/or the fetus. By comprehensively reviewing these changes, healthcare professionals can counsel and educate their patients and ensure they receive appropriate care and intervention when necessary [3]. The abdomen (trunk) is particularly prone to skin changes due to the mechanical stretching from the growing uterus and the localised effects of hormonal shifts [3].

## Review

**Figure 1:**
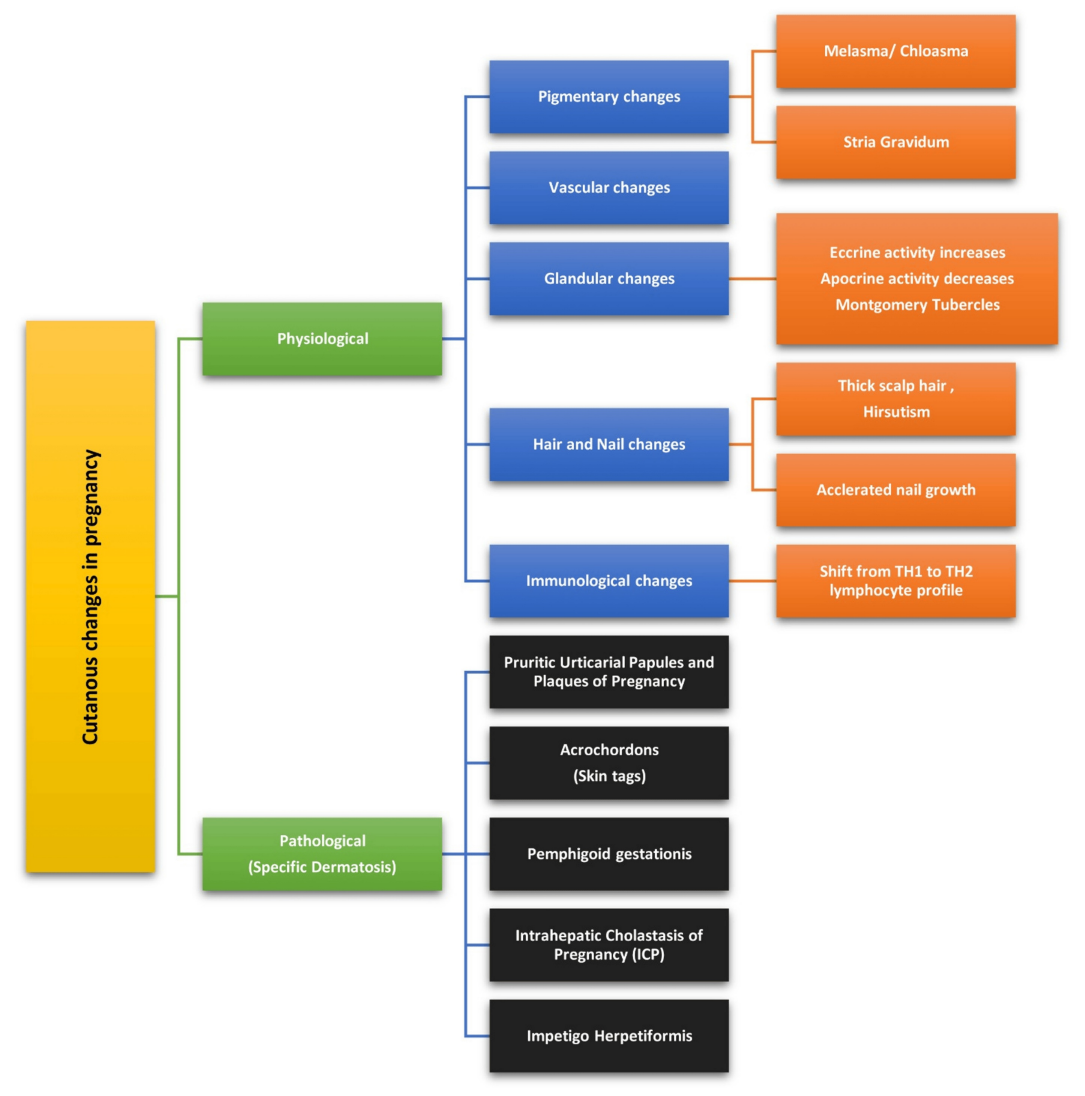
Classification of various changes in pregnancy Image credits :  Dr. Shreya N. Gupta

The various cutaneous conditions can be further classified into Physiological and Pathological or Specific changes as illustrated in Figure 1.

Physiological cutaneous changes in pregnancy

Hyperpigmentation

Hyperpigmentation is among the most common skin changes during pregnancy, affecting up to 90% of women [4-8]. Melasma, often referred to as the "mask of pregnancy," manifests as brown patches on the face, particularly on the cheeks and forehead. This condition, also known as chloasma, is specifically associated with the mask-like hyperpigmentation seen in pregnant women. Another form of hyperpigmentation, linea nigra, appears as a dark vertical line running from the pubic area to the umbilicus. Additionally, peri-areolar hyperpigmentation occurs around the nipples. The increased melanin production responsible for these changes is attributed to elevated levels of hormones, such as estrogen, progesterone, and melanocyte-stimulating hormone, during pregnancy. Placental lipids may also contribute to this effect. While most hyperpigmentation fades after delivery, certain areas, like the linea nigra and areolas, often do not return to their original color. Melasma may persist for several years in some women. Limiting sun exposure, using sunscreen, and wearing protective clothing are recommended to minimize hyperpigmentation. Topical treatments, including kojic acid, liposomal aloe vera, and nicotinamide, may be considered safe integrative approaches. However, conventional treatments such as hydroquinone and retinoids are controversial and generally avoided during pregnancy [4-8].

Striae Gravidarum (Stretch Marks)

Striae gravidarum, commonly known as stretch marks, are common during pregnancy, affecting up to 90% of pregnant women. They typically appear on the abdomen, breasts, hips, and thighs as reddish-purple, linear atrophic bands that develop perpendicular to the skin's tension lines as they rapidly stretch to accommodate the growing fetus. Hormonal changes, rapid weight gain, and genetic predisposition cause stretch marks. Younger women, those with higher body mass indices, and those carrying larger babies are at higher risk of developing severe stretch marks. While stretch marks may initially cause some itching and burning sensations, they pose no health risks and do not compromise the body's ability to function normally. Over time, they fade to silvery-white or appear as flesh-coloured streaks but often remain as permanent skin discolorations [9,10]. No proven methods prevent stretch marks during pregnancy [9]. However, maintaining a healthy weight, staying hydrated, and using moisturizing creams during pregnancy may help reduce the disease severity. After delivery, topical retinoids, laser treatments (585 nm Pulse dye laser, 1064-nm long-pulsed neodymium-doped yttrium aluminium garnet (Nd:YAG) laser, 2940-nm variable square pulse erbium yttrium aluminium garnet (Er:YAG) laser or ablative fractional CO2 laser), or chemical peels can be used to lighten the appearance of stretch marks, but they are not always effective and may carry some risks [9-11]. Striae gravidarum is a common and normal consequence of pregnancy that typically fades over time but sometimes may persist as permanent marks on the skin. While treatments are available, the best approach is to manage expectations and focus on overall skin health during and after pregnancy.

Vascular Changes

Pregnancy induces significant vascular changes to support the growing fetus. Maternal blood volume begins to increase early in pregnancy (around 6-8 weeks of gestation), eventually reaching a maximum of approximately 50% greater than the non-pregnant state. Cardiac output also rises, driven by increases in both heart rate and stroke volume, resulting in a 30-50% increase during pregnancy, with most of this change occurring within the first eight weeks of gestation [12]. Despite these increases in blood volume and cardiac output, blood pressure typically does not rise due to a general decrease in systemic vascular resistance, particularly in the uterine vasculature. Uterine blood flow is estimated to increase by 30 to 50 times compared to the non-pregnant state. Early in pregnancy, cytotrophoblast invasion into the endometrium initiates vascular remodeling, leading to the formation of sinuses that eventually develop into placental villi. By the second trimester, the myometrial spiral arteries are transformed from high-resistance, coiled vessels into dilated, low-resistance vessels [13]. These profound vascular adaptations are driven primarily by hormonal changes, especially estrogen and progesterone, and are essential for supporting the growing fetus. Clinically, these vascular changes manifest as common signs such as spider angiomas (nevi aranei) and palmar erythema. Additional symptoms include flushing of the skin and temporary edema of the face, hands, and feet. Erythema of the vestibule and vagina (Jacquemier-Chadwick sign) and the bluish discoloration of the cervix (Goodell sign) are early diagnostic indicators of pregnancy [14].

Pathological dermatosis in pregnancy

PUPPP (Pruritic Urticarial Papules and Plaques of Pregnancy)

Pruritic Urticarial Papules and Plaques of Pregnancy (PUPPP) are characterized by an intensely itchy rash that typically begins on the abdomen, often within stretch marks, and may spread to the thighs, legs, back, buttocks, arms, and breasts. The rash consists of red, raised, hive-like papules and plaques, while the face, palms, and soles are generally unaffected [15]. The exact cause of PUPPP is not fully understood, but it is thought to be related to the rapid stretching of the skin during pregnancy. Theories include an immune reaction to fetal cells, hormonal changes, and damage to the connective tissue in the skin. PUPPP is more common in first-time pregnancies, multiple gestations, pregnancies with rapid weight gain, or pregnancies involving large babies, typically appearing in the third trimester [15]. PUPPP is primarily diagnosed based on the characteristic rash and its distribution. Diagnostic tests may be conducted to rule out other pregnancy-related skin conditions, such as pemphigoid gestationis. Management focuses on symptomatic relief through the use of topical corticosteroids, oral antihistamines, and other antipruritic measures. The rash usually resolves spontaneously within 4-6 weeks after delivery [16]. PUPPP is a benign condition that poses no risk to the mother or fetus. The rash typically resolves without scarring, and recurrence in subsequent pregnancies is uncommon.

Acrochordon (Skin Tags)

Skin tags are small, benign skin growths that commonly develop during pregnancy due to hormonal changes and increased skin friction. These growths consist of loose collagen fibers and blood vessels enclosed by the skin and typically appear in areas where skin folds and creases. While skin tags are generally harmless and do not require treatment, some women may opt to have them removed for cosmetic reasons [17]. The increased likelihood of developing skin tags during pregnancy can be attributed to several factors. Hormonal fluctuations, particularly in estrogen and progesterone, can stimulate the formation of skin tags. Additionally, as the abdomen and other areas expand, increased skin-on-skin contact and friction promote the growth of skin tags. Weight gain during pregnancy can also create more skin folds and creases, which are common sites for skin tag development [18]. Although skin tags often resolve on their own after pregnancy, there are several options for removal, including cryotherapy (freezing), cauterization (burning), and excision (cutting out). These procedures should be performed by a dermatologist or a qualified healthcare provider to minimize risks such as bleeding and scarring. Over-the-counter skin tag removal products should be avoided, as they carry a higher risk of complications [19]. While it may not be possible to prevent skin tags entirely, wearing loose, breathable clothing can help reduce irritation and discomfort [19].

Pemphigoid Gestationis

Pemphigoid gestationis (PG), also known as herpes gestationis, is a rare autoimmune skin disorder that typically arises during pregnancy, most commonly in the second or third trimester. It is marked by intensely itchy, red papules and blisters that usually begin around the navel and can spread to the abdomen, trunk, and occasionally other areas of the body. PG is caused by autoantibodies targeting a protein called BP180 (also known as BPAG2 or collagen XVII), which is located in the basement membrane zone of the skin. The precise trigger for this autoimmune response remains unclear. Symptoms often improve toward the end of pregnancy, but flare-ups are common around the time of delivery [20-22]. In most cases, the skin lesions heal within weeks to months after childbirth, though PG can persist for years in some women. The condition may also recur with subsequent pregnancies, oral contraceptive use, or menstruation. Diagnosis is based on the characteristic skin lesions and confirmed through a biopsy showing a linear band of C3 at the basement membrane zone, as well as direct immunofluorescence. PG can be clinically confused with other pruritic eruptions of pregnancy, such as pruritic urticarial papules and plaques of pregnancy (PUPPP). Treatment aims to relieve itching and prevent blister formation, often through the use of topical or systemic corticosteroids. Antihistamines may also be employed. The lowest effective dose of medication is used to minimise risks to both the mother and fetus. While most fetuses remain unaffected, PG can lead to complications such as premature delivery and infants being small for gestational age. In rare instances, neonates may develop transient skin lesions similar to those seen in the mother [20-24].

Intrahepatic Cholestasis of Pregnancy (ICP)

Intrahepatic Cholestasis of Pregnancy (ICP) is a liver disorder that typically develops during pregnancy, usually in the late second or early third trimester. It is characterised by the accumulation of bile acids in the blood due to impaired bile flow from the liver. The primary symptom of ICP is intense itching, often beginning on the palms and soles before spreading to other areas of the body. Occasionally, jaundice (noticeable in the skin and sclera) may also occur [25]. ICP is thought to arise from a combination of genetic, hormonal, and environmental factors. Variations in several genes involved in bile acid synthesis and transport, particularly *ABCB4 *(ATP-binding cassette, subfamily B, member 4), which facilitates the transport of phospholipids into the canalicular lumen in the presence of bile salts, increase the risk of developing ICP. This gene encodes a protein that moves specific fats (phospholipids) across liver cell membranes, releasing them into bile-a digestive fluid. Additionally, pregnancy hormones like estrogen and progesterone further impair the liver's ability to transport bile acids effectively [25]. ICP poses significant risks to the fetus, including an increased likelihood of premature delivery, respiratory distress, meconium aspiration, fetal distress, and stillbirth. The risk of these complications correlates with the level of bile acids in the mother's blood. Management of ICP focuses on symptom relief and monitoring bile acid levels to plan delivery timing. Medications like ursodeoxycholic acid (UDCA) are commonly used, and delivery is typically recommended between 36-39 weeks, with earlier delivery considered in severe cases. While maternal outcomes are generally favorable, with symptoms usually resolving after delivery, ICP can recur in up to 90% of future pregnancies. Therefore, close monitoring and management by both the obstetrician and hepatologist are crucial to minimizing fetal risks [25].

Impetigo Herpetiformis

**Figure 2:**
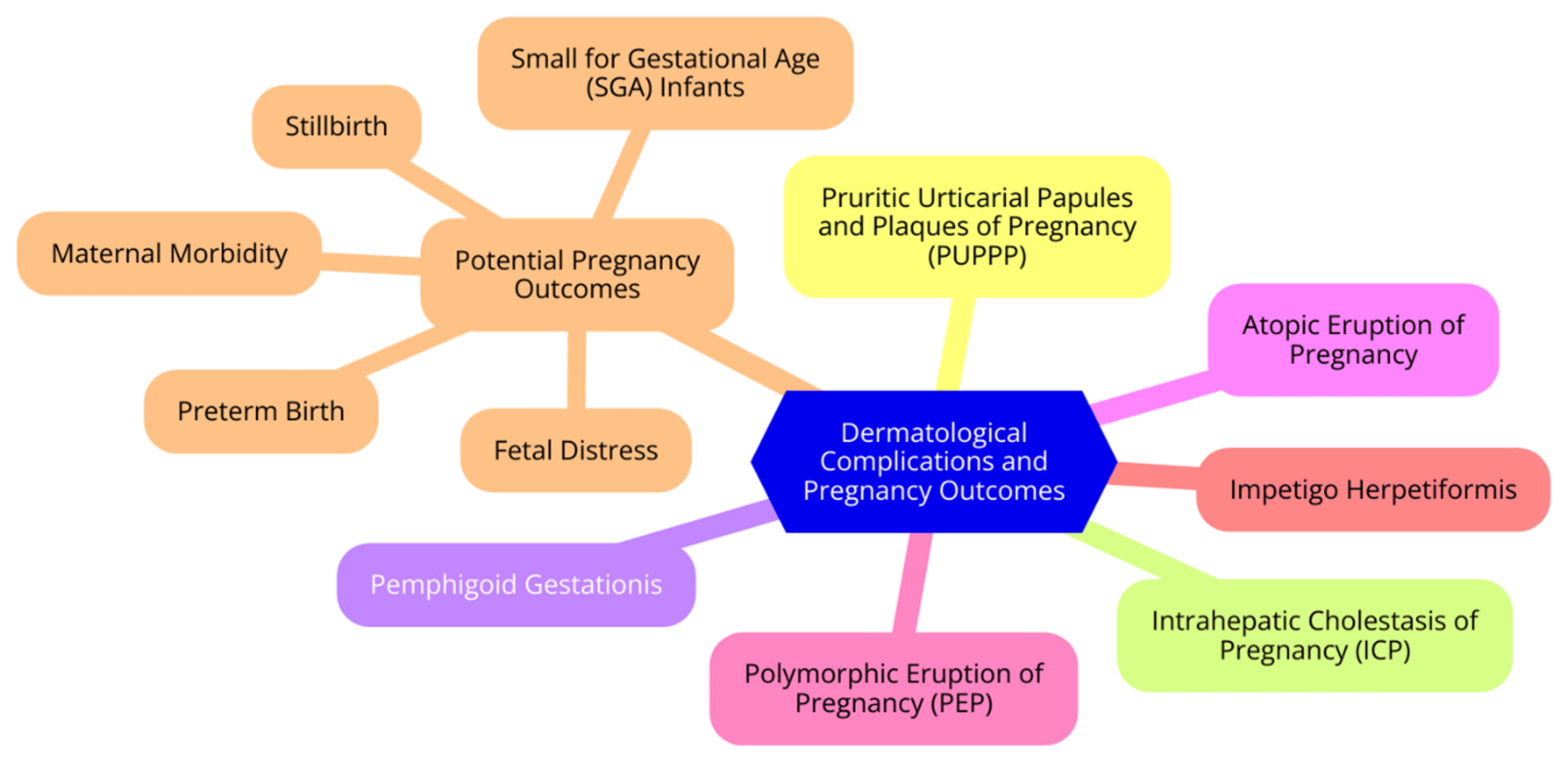
Dermatological complications and pregnancy outcomes Image Credit: Dr Shreya N. Gupta

Impetigo herpetiformis (IH) is a rare and potentially life-threatening dermatosis that typically occurs during the third trimester of pregnancy and may recur in subsequent pregnancies [26]. The condition is characterized by erythematous patches with marginally grouped sterile pustules, which primarily appear in flexural regions. These lesions can erode, crust, and may even become impetiginized as they spread centrifugally. IH is sometimes associated with hypoparathyroidism and hypocalcemia [27]. Systemic symptoms of IH can include nausea, vomiting, fever, chills, diarrhea, hypovolemic shock, seizures, and general malaise [28]. Although there are no established treatment guidelines, it is critical to promptly correct fluid and electrolyte imbalances, particularly hypovolemia, hypocalcemia, and low vitamin D levels [29]. Systemic corticosteroids are the mainstay of treatment for IH. In mild to moderate cases, an initial dose of 15-30 mg daily is common, which can be increased to 40-60 mg or even up to 80 mg daily if necessary. While corticosteroids are the preferred therapy, their excessive use has been associated with risks such as intrauterine growth retardation (IUGR) and cleft palate in newborns [30]. Cyclosporine is another treatment option, administered at a dosage of 2-7.5 mg/kg/day, with variable outcomes. Once cyclosporine is initiated, corticosteroids can be tapered [31]. Biologics like infliximab and adalimumab are classified as pregnancy category B drugs. The National Psoriasis Foundation considers infliximab one of the best therapies for IH; however, this contrasts with guidelines from the European Academy of Dermatology and Venereology, which does not recommend the use of adalimumab or infliximab during pregnancy [32]. Phototherapy, specifically narrowband UVB (NBUVB), is considered one of the safest treatment modalities and may be added to therapy when an adequate response cannot be produced with corticosteroids [33]. Dermatological complications during pregnancy and their possible outcomes are illustrated in Figure 2.

Dermatological complications and pregnancy outcomes

Impact on Maternal Health

During pregnancy, various skin changes can occur, affecting up to 90% of women. Melasma is a common condition. Limiting sun exposure and using sunscreen are crucial to prevent its worsening [4]. Stretch marks appear in about 90% of pregnant women. Although they are generally benign and do not require treatment, they can be unsightly [4]. PUPPP can significantly affect a woman's quality of life due to severe itching, often requiring antihistamines and topical steroids [4,34]. Certain pregnancy-specific dermatoses like pemphigoid gestationis, impetigo herpetiformis, and intrahepatic cholestasis of pregnancy can pose risks to both the mother and fetus if not managed properly. These conditions necessitate close monitoring by obstetricians and dermatologists to minimize potential complications [28-31]. While most pregnancy-related skin changes are harmless, conditions such as PUPPP and specific dermatoses can profoundly impact maternal health and quality of life. Therefore, careful monitoring and treatment by healthcare professionals are essential to ensure optimal outcomes for both the mother and baby.

Implications for fetal development

While common, skin changes that occur during pregnancy do not typically have direct implications for fetal development [35].

Dermatological Drugs During Pregnancy and Lactation

Treatment of cutaneous conditions in pregnancy is elective. Some drugs used by dermatologists for treating such conditions may cause harm to the growing fetus. For this reason, a general reluctance exists between the patient and the treating physician to prescribe oral medications during pregnancy [36]. A list of preferred drugs in pregnancy has been adapted from Wolverton and Wu (2021), Box 65.1 (Table 1) [37].

**Table 1:** Preferred drugs in pregnancy Source: Wolverton and Wu (2021), Box 65.1 [37].

Sr no	Class	Drug name
1	Analgesics	Acetaminophen
2	Anaesthetics	Lidocaine
3	Antibacterial Agents	Erythromycins, Penicillins
4	Anti fungal Agents	Fluconazole, Nystatin^b, ^Clotrimazole^b, ^Miconazole^b^
5	Antihistamines	Chlorpheniramine^a^
6	Antiviral Agents	Acyclovir
7	Anti-scabetic Agents	Permethrin: topical
8	Corticosteroids	Oral: Avoid high doses first trimester; use prednisone, prednisolone or methylprednisolone, Topical: mild/moderate preferred; avoid potent/ very potent topical corticosteroids in large amounts
9	Miscellaneous Topical Antiacne Products	Azelaic acid, Benzoyl peroxide, Erythromycin

General management of cutaneous conditions occurring in pregnancy

Safe-to-use Topical Treatments in Various Cutaneous Conditions in Pregnancy

Topical antibacterial agents like mupirocin or clindamycin can treat or prevent skin infections during pregnancy. While side effects are uncommon, they may include skin irritation, dryness, or redness. Similarly, anti fungal agents like clotrimazole, ketoconazole, and terbinafine are used for fungal skin conditions. These topical anti-fungals are generally well-tolerated, with side effects such as stinging, itching, or redness occurring infrequently [38]. Benzoyl peroxide creams, gels, and washes can be effective for acne management. However, common side effects include dryness, peeling, and skin irritation. Topical retinoids are also used to treat acne but may cause intense burning, stinging, dryness, redness, or peeling. Salicylic acid products can help with acne and warts, and commonly cause mild skin irritation [39]. Corticosteroids are the main topical anti-inflammatory medications used during pregnancy to reduce swelling, itching, and redness from conditions like eczema or allergic reactions. While effective, corticosteroids should be used cautiously on sensitive areas like the face, as prolonged use can cause skin thinning, striae, and acne-like eruptions. Usage for appropriate indication and monitoring for side effects is important, especially with corticosteroids on sensitive areas [40-41].

Safe-to-use Systemic Treatments in Various Cutaneous Conditions in Pregnancy

Data on the safety and efficacy of systemic therapies like chemotherapy, hormonal therapy, and targeted therapy during pregnancy is limited but available. Certain systemic medications should be avoided during pregnancy, such as tamoxifen (hormonal therapy) and herceptin/trastuzumab (targeted therapy) [42]. Cyclosporine was identified as the first-choice long-term systemic treatment for atopic dermatitis in women during preconception, pregnancy, and breastfeeding. There was no consensus on second-choice systemic medications for atopic dermatitis during preconception or pregnancy, though dupilumab and azathioprine were deemed suitable during breastfeeding. Medications like Janus kinase (JAK) inhibitors, methotrexate, and mycophenolate mofetil should generally be avoided by women during preconception, pregnancy, and breastfeeding [43]. Table 1 lists the drugs preferred over other drugs in their class in gestation. The use of systemic therapies during pregnancy requires careful consideration of the available evidence on safety and efficacy [44].

Non-Pharmacological Interventions

Non-pharmacological interventions for managing common skin conditions can be very effective when used alongside drug therapy. Specific interventions include patient education, trigger avoidance, and psychological support for interrupting the itch-scratch cycle [45]. Individual interventions comprise educational programs, psychosocial support, and techniques like motivational interviewing. Non-pharmacological treatments are especially indicated for chronic skin diseases, though some acute conditions can also benefit from such treatments [46]. These non-drug approaches are best used as adjuncts to drug therapy, not substitutes, to optimise outcomes and quality of life. Interventions, are best utilised when tailored to patients based on their needs and disease factors [47].

## Conclusions

In conclusion, the cutaneous changes that occur during pregnancy, particularly on the face and trunk, directly result from the complex interplay of hormonal, mechanical, and immunological factors. While often benign, these changes can significantly impact a woman's physical comfort and psychological well-being. Understanding the underlying mechanisms and manifestations of these cutaneous alterations is crucial for providing comprehensive prenatal care. By recognizing common conditions such as hyperpigmentation, striae gravidarum, and vascular changes, as well as rarer dermatological issues like pemphigoid gestationis and intrahepatic cholestasis of pregnancy, healthcare providers can reassure and better manage these patients. Furthermore, emphasizing preventive measures, effective management strategies, and patient education can mitigate the adverse effects of these skin changes. This review highlights the cutaneous changes that occur during pregnancy and their impact on the well-being of expectant mothers and can help healthcare providers recognise and manage such conditions.
